# Single Molecule Fluorescence In Situ Hybridization Using RNAscope to Study Hematopoietic and Vascular Interactions in the Zebrafish Embryo and Larva

**DOI:** 10.21769/BioProtoc.5269

**Published:** 2025-04-05

**Authors:** Léa Torcq, Anne A. Schmidt

**Affiliations:** 1 Institut Pasteur, Université Paris Cité, CNRS UMR3738, Department of Developmental and Stem Cell Biology, Paris, France; 2 Sorbonne Université, Collège doctoral, Paris, France

**Keywords:** Hematopoiesis, cmyb, smFISH, RNAscope, Confocal microscopy, Imaris, Zebrafish embryo, Zebrafish larva

## Abstract

Our goal is to understand how hematopoietic stem cell precursors emerge from vessels and to visualize their settling in developmental and more definitive niches that will persist in the adult. For this, we use as a biological model the zebrafish, which offers invaluable advantages owing to its transparency and small size, allowing high-resolution imaging and investigations of the entire animal. In vertebrate species, precursors of hematopoietic stem cells emerge from arterial vessels, mainly from the ventral side of the dorsal aorta. From there, they can either reside in the underlying vascular niche and/or pass through the vein to enter the blood circulation and conquer the caudal hematopoietic tissue, a functional equivalent to the fetal liver in mammals. Here, we provide experimental details of a protocol we have recently optimized to identify, based on mRNA in situ hybridization, precursors of hematopoietic stem cells while still embedded in the aortic wall (at the embryonic stage) as well as when they reside in specific niches a few days after emergence (at the early larval stage). Our experimental approach uses RNAscope technology, which allows combining high-sensitivity mRNA detection with high-resolution fluorescence confocal imaging to achieve spatial transcriptomics. Importantly, the small size of the probes allows better penetration inside tissues, which is a significant improvement in comparison to long mRNA probes; this is an invaluable advantage for reaching deeply embedded niches such as the ones of the pronephros region in the larva and, in addition, provides an increased signal-to-noise ratio.

Key features

• Optimized protocol for high detection sensitivity of mRNAs expressed in the zebrafish embryo and larva, in combination with high spatial resolution using fluorescence confocal microscopy.

• In toto visualization and quantification, in zebrafish larvae, of hematopoietic populations in their niches, including niches deeply embedded into internal organs.

• Possible upgrades for multiplexing of mRNA detection.

## Graphical overview


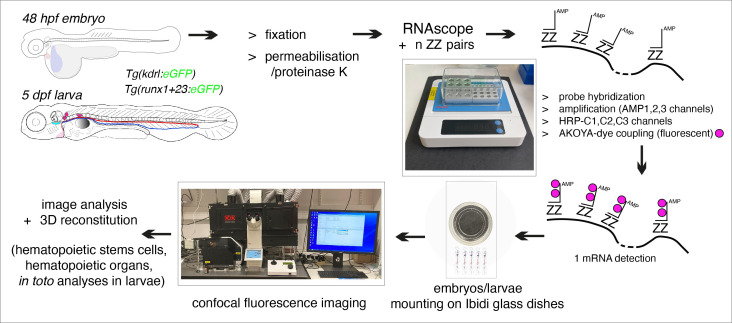


**Flowchart of the principal steps of the RNAscope protocol applied to fluorescent transgenic zebrafish embryos and larvae**

## Background

Blood and immune cells of the vertebrate adult body are born during a very narrow time window of embryonic development. More precisely, the cells giving birth to blood and immune cells are hematopoietic stem cell precursors that emerge from vessels and, more specifically, arterial components in embryos [1–4]. Not all cells constituting the aortic wall are programmed to become competent for giving birth to hematopoietic precursors; only a restricted subset of cells, collectively named the hemogenic endothelium (HE), possess this transient property, as initially suggested by studies in avian and mice [5,6]. Hematopoietic stem cell precursors emerge essentially from the aortic floor, according to a process dynamically revealed by high spatially and temporally resolved confocal imaging in the zebrafish embryo (referred to as the endothelial-to-hematopoietic transition, EHT; see [4,7]). This animal model offers unique advantages for visualizing cellular dynamics in vivo and, in the more specific context of the EHT, unveiling the morphodynamic heterogeneity of EHT-undergoing cells [8] that may lead to differentially fated cells, after their release from the aortic wall. Another invaluable advantage of the zebrafish model for studying developmental hematopoiesis is the possibility to use imaging-based cell fate tracing approaches to follow hematopoietic stem cell precursors from their emergence site to specific organs serving as niches [9]; these niches will host hematopoietic stem and progenitor cells (HSPCs) more or less transiently for multiplication, differentiation, and maintenance. In the zebrafish, during early development, these niches are the caudal hematopoietic tissue (CHT) in the tail region [10,11], constituted by the vein plexus (considered to be the functional equivalent of the fetal liver in mammals), the thymus, and the pronephros region (the kidney marrow will then become the definitive niche in the adult fish, the functional equivalent to the bone marrow in mammals). Overall, the relatively small size of the zebrafish embryo and early larva, together with high-resolution confocal imaging at the cellular and sub-cellular levels, offer unique opportunities to obtain an extended cartography of HSPC populations homing in hematopoietic organs and more local niches. In addition, with the advent of single-cell technologies that provide gene expression signatures of unique cell populations, we can improve the visualization of hematopoietic sub-populations in niches by combining mRNA detection in situ (in whole-mount embryos and larvae) with high-resolution imaging, thus unveiling subtle microenvironmental peculiarities.

Here and in this context, we build on previous pioneering work using the zebrafish embryo as a model [12,13] and describe a methodological pipeline including the RNAscope technology and 3D image analysis to reconstitute the homing of HSPCs in developing hematopoietic organs in the entire zebrafish embryo and larva. This protocol can be translated to other organs and organisms amenable to high-resolution imaging approaches.

## Materials and reagents

**Biological materials**

1. *Tg(kdrl:eGFP)* [14]

2. *Tg(runx1+23:eGFP*) [14] (also published in [11]). Importantly, to maximize signal and limit mosaicism (increased by transgenerational silencing and/or loss of transgene), the experiments performed in this work were obtained using the F1 generation

Adult fish lines were maintained on 14/10 h light/dark cycles. Embryos were collected in Volvic water containing methylene blue and PTU to prevent pigmentation and raised at 28.5 °C until use. Embryos were all manually dechorionated, between 35 and 48 hpf. Embryos and larvae used for imaging were staged at 48–50 hpf and 5 dpf, precluding sex determination

**Reagents**

1. Methylene blue (Sigma-Aldrich, catalog number: M4159)

2. N-Phenylthiourea (PTU) (Sigma-Aldrich, catalog number: P7629)

3. Tricaine methanesulfonate (MS-222) (Sigma-Aldrich, catalog number: A5040)

4. Formaldehyde (FA), 10% stock solution (Electron Microscopy Sciences, catalog number: 15712)

5. Tween 20 (Sigma-Aldrich, catalog number: P7949)

6. PBS 10× powder concentrate (Fisher Scientific, catalog number: BP665-1)

7. 100% Methanol (MeOH) (Sigma-Aldrich, catalog number: 34860-1L-R)

8. mQH_2_O

9. Proteinase K (glycerol stock at 20 mg/mL) (Ambion, catalog number: 10259184)

10. Multiplex Fluorescent Reagent kit v2 (ACD BioTechne, catalog number: 323100; https://acdbio.com/rnascope-multiplex-fluorescent-v2-assay). Includes H_2_O_2_, probe diluent (PD), wash buffer (WB), AMP1, AMP2, and AMP3 buffers, HRP-C1, HRP-C2, and HRP-C3 reagents, TSA buffer, and HRP blocker. Note that the supplier does not provide information on reagent concentrations. Note also that the kit provides enzymes for tissue digestion [none of them were tested because we privileged proteinase K for whole-mount in situ hybridization (WISH), which uses long anti-sense nucleotide probes incubated with entire embryos/larvae]

11. RNAscope^TM^ probe Dr-myb (ACD BioTechne, catalog numbers: 558291, 558291-C2, 558291-C3, for the three channels C1, C2, and C3, respectively)

12. RNAscope^TM^ negative control probe DapB (of *Bacillus subtilis* strain) (ACD BioTechne, catalog numbers: 310043, 310043-C2, 310043-C3 for the three channels C1, C2, and C3, respectively)

13. OPAL-480 (Akoya Biosciences, catalog number: FP1500001KT)

14. OPAL-570 (Akoya Biosciences, catalog number: FP1488001KT)

15. OPAL-690 (Akoya Biosciences, catalog number: FP1497001KT)

16. Low melting agarose (Promega, catalog number: V2111)

17. Volvic water (Dutscher, catalog number: 067507)

*Note: The composition of Volvic water is as follows: bicarbonates (74 mg/L), silica (32 mg/L), chlorites (15 mg/L), calcium (12 mg/L), sodium (12 mg/L), sulfates (9 mg/L), magnesium (8 mg/L), nitrates (7.3 mg/L), and potassium (6 mg/L). Although not tested, we propose as a possible alternative the use of embryo medium, which may be more commonly used in other laboratories. The composition of the embryo medium should be NaCl (292 mg/L), KCl (13 mg/L), CaCl_2_ (44 mg/L), and MgSO_4_ (81 mg/L).*

**Solutions**

1. 50× PTU solution (see Recipes)

2. 25× Tricaine (see Recipes)

3. 10× PBS (reconstituted from powder) (see Recipes)

4. 1× PBS (see Recipes)

5. 1× PBS/0.1% Tween 20 (PBST) (see Recipes)

6. Formaldehyde fixation (see Recipes)

7. 25% MeOH (see Recipes)

8. 50% MeOH (see Recipes)

9. 75% MeOH (see Recipes)

10. Dilution of probe in -C2 channel (1/50) (see Recipes)

11. Dilution of probe in -C3 channel (1/50) (see Recipes)

12. Dilution of probes in -C2 and -C3 channels (1/50 each) (see Recipes)

13. Dilution of probes in -C1, -C2, and -C3 channels (see Recipes)

14. Dilution of probes in -C1 and -C2 or -C3 channels (see Recipes)

15. 1× WB (RNAscope wash buffer) (see Recipes)

16. Dilution of OPAL dyes (see Recipes)

17. 2% low melting agarose (see Recipes)

18. Embryo collection medium (see Recipes)

19. Anesthetic solution (see Recipes)

20. Proteinase K solution (see Recipes)

21. 1% low melting agarose diluted in PBST (see Recipes)

**Recipes**

**1. 50× PTU solution**

The PTU solution must be prepared and kept in the dark (prepare and store the solution in an opaque container, for example, a bottle wrapped in aluminum foil). The PTU 50× solution is prepared by dissolving the powder in mQH_2_O at 50 °C with magnetic steering overnight (O/N) under the chemical hood. The solution can be kept at room temperature (RT) for several weeks.

**Table:** 

Reagent	Final concentration	Quantity or Volume
N-Phenylthiourea powder	1,500 mg/L	750 mg
mQH_2_O	n/a	500 mL
Total	n/a	500 mL

**2. 25× Tricaine**

The tricaine 25× solution is prepared by dissolving the powder upon magnetic steering O/N, under the chemical hood, at RT. Subsequently, it is aliquoted in 15 mL Falcon tubes and stored frozen at -20 °C until use.

**Table:** 

Reagent	Final concentration	Quantity or Volume
Tricaine powder	4 g/L	1 g
mQH_2_O	n/a	250 mL
Total	n/a	250 mL

**3. 10**× **PBS (reconstituted from powder)**

**Table:** 

Reagent	Final concentration	Quantity or Volume
PBS 10× powder concentrate (1 pack)	10×	98.9 g
mQH_2_O	n/a	0.95 L
Total	n/a	1 L

**4. 1**× **PBS**

**Table:** 

Reagent	Final concentration	Quantity or Volume
PBS 10×	1×	100 mL
mQH2O	n/a	900 mL
Total	n/a	1 L

**5. 1**× **PBS/0.1% Tween 20 (PBST)**

**Table:** 

Reagent	Final concentration	Quantity or Volume
PBS	1×	49.750 mL
Tween 20	0.1%	0.250 mL
Total	n/a	50 mL

**6. Formaldehyde fixation**

Volume for 10 Eppendorf tubes of 1.5 mL, each containing 15–20 embryos/larvae in 1 mL of fixation solution.

**Table:** 

Reagent	Final concentration	Quantity or Volume
10% formaldehyde	4%	4 mL
PBST	n/a	5.6 mL
Tricaine 25×	1×	0.4 mL
Total	n/a	10 mL

**7. 25% MeOH**

**Table:** 

Reagent	Final concentration	Quantity or Volume
100% MeOH	25%	2.5 mL
mQH_2_O	n/a	7.5 mL
Total	n/a	10 mL

**8. 50% MeOH**

**Table:** 

Reagent	Final concentration	Quantity or Volume
100% MeOH	50%	5 mL
mQH_2_O	n/a	5 mL
Total	n/a	10 mL

**9. 75% MeOH**

**Table:** 

Reagent	Final concentration	Quantity or Volume
100% MeOH	75%	7.5 mL
mQH_2_O	n/a	2.5 mL
Total	n/a	10 mL

**10. Dilution of probe in -C2 channel (1/50)**

Probes can be synthesized by the supplier in three channels: C1, C2, and C3. Probes in different channels have the same RNA recognition sequence (ZZ probe lower part) but a different amplification complex recognition sequence (ZZ probe upper part) that allows them to be combined simultaneously for multiplexing. Probes in specific channels can be found in the catalog of the supplier and, if not, they need to be designed in the appropriate channel (in this case, the DNA sequence and accession number need to be communicated to the design department of the supplier). All probes in C1 are delivered already diluted 1/50 in the probe diluent (PD). All other probes need to be diluted 1/50 in PD.

**Table:** 

Reagent	Final concentration	Quantity or Volume
Probe C2	1/50	2 μL
Probe diluent	n/a	98 μL
Total	n/a	100 μL

**11. Dilution of probe in -C3 channel (1/50)**

**Table:** 

Reagent	Final concentration	Quantity or Volume
Probe C3	1/50	2 µL
Probe diluent	n/a	98 µL
Total	n/a	100 µL

**12. Dilution of probes in -C2 and -C3 channels (1/50 each)**

All probes can be co-incubated with biological material if in different channels (see *probe hybridization* in Section B). However, it is recommended to test probes individually to evaluate their respective background. It is also recommended to test individual control probes (and in combination, if pertinent), in all channels corresponding to the probe(s) used.

**Table:** 

Reagent	Final concentration	Quantity or Volume
Probe C2	1/50	2 µL
Probe C3	1/50	2 µL
Probe diluent	n/a	96 μL
Total	n/a	100 µL

**13. Dilution of probes in -C1, -C2, and -C3 channels**

**Table:** 

Reagent	Final concentration	Quantity or Volume
Probe C1	1/50	96 µL
Probe C2	1/50	2 µL
Probe C3	1/50	2 µL
Total	n/a	100 µL

**14. Dilution of probes in -C1 and -C2 or -C3 channels**

**Table:** 

Reagent	Final concentration	Quantity or Volume
Probe C1	1/50	98 µL
Probe C2 (or C3)	1/50	2 µL
Total	n/a	100 µL

**15. 1**× **WB (RNAscope wash buffer)**

Before dilution of the WB, make sure that the 50× solution is homogenized. If necessary, warm up at approximately 30 °C for 10–15 min and shake before dilution.

**Table:** 

Reagent	Final concentration	Quantity or Volume
WB 50×	1×	1 mL
mQH_2_O	n/a	49 mL
Total	n/a	50 mL

**16. Dilution of OPAL dyes**

This recipe is applicable to each individual OPAL dye, here with OPAL-570, which is used in Figures 3–7. The total volume will depend on how many samples are assayed (the calculation below is for one sample).

**Table:** 

Reagent	Final concentration	Quantity or Volume
OPAL-570	1/500	1 µL
TSA buffer	n/a	499 µL
Total	n/a	500 µL

**17. 2% low melting agarose**

**Table:** 

Reagent	Final concentration	Quantity or Volume
Low melting agarose	2% (w/v)	1 g
PBS	1×	50 mL

**18. Embryo collection medium**

Volvic source water + 280 μg/L methylene blue + PTU (0.003%, final concentration).

**19. Anesthetic solution**

Use tricaine at 160 μg/mL final concentration [diluted into Volvic source water, from a 4 mg/mL stock (mQH_2_O)]. Kept aliquoted at -20 °C until use.

**20. Proteinase K solution**

Diluted at 1/2,000 in PBST and from a glycerol stock solution at 20 mg/mL kept at -20 °C.

**21. 1% low melting agarose diluted in PBST**

Embedding for imaging is done in 1% low melting agarose diluted in PBST. After solidification of the agarose, 1 mL of PBS is added to solidified agarose to avoid dehydration during acquisitions, which also allows maintaining the dishes if kept at 4 °C until imaging (for a maximum of 2 days).

*Note: Although not tested, 0.02% NaN_3_ may be added to the PBS to prevent contamination.*

**Laboratory supplies**

1. Glass bottom 60 μ-dish, 35 mm high (Ibidi, catalog number: 81156)

2. Petri dishes 100 × 20 mm (Dutscher, catalog number: 353003)

3. Dumont #55 forceps (Fine Science Tools, catalog number: 11255-20)

4. Dumont #55F forceps (Fine Science Tools, catalog number: 11252-00)

5. Microtube Eppendorf tubes, 1.5 mL (Dutscher, catalog number: 033290)

6. Microtube Eppendorf tubes, 2 mL (Dutscher, catalog number: 033297)

7. 25G-needles, Microlance 3 (Dutscher, catalog number: 300600)

8. Microloader Eppendorf long plastic tips (Dutscher, catalog number: 034903)

9. 15 mL Corning polypropylene tubes (Dutscher, catalog number: 4307919)

10. 50 mL Corning polypropylene tubes (Dutscher, catalog number: 430828)

## Equipment

1. Binocular and fluorescence lamp for fluorescent transgenic embryo/larvae selection

2. Rocking platform for washes (Stuart Gyratory rocker SSL3, catalog number: Z654515)

3. Dry heating block for temperature-controlled hybridization (Thermo Scientific, catalog number: 88871003)

4. Confocal spinning disk station [the entire imaging station from Andor (Oxford Instruments), comprising a spinning disk confocal system (CSU-W1), a Leica DMi8 fluorescence inverted microscope, CMOS cameras, and 40× water immersion objective, is described in [7,8].

## Software and datasets

1. Imaris file converter, free proprietary software (version 10.1.0), https://imaris.oxinst.com/microscopy-imaging-software-free-trial#file-converter

2. Imaris Viewer, free proprietary software (version 9.9.1), https://imaris.oxinst.com/imaris-viewer

3. Imaris, license required (Imaris for Cell Biologists package, version 10.1.0), https://imaris.oxinst.com/products/imaris-for-cell-biologists

4. Napari, free open-source software (version 0.4.12), https://napari.org/stable/index.html

5. ImageJ (FIJI), free open-source software (version 2.3.0), https://imagej.net/software/fiji/downloads

6. Icy, free open-source software (version 2.5.2.0), https://icy.bioimageanalysis.org/

7. R (version 4.2.3, 2023-03-15), https://www.r-project.org/

8. R Studio, https://posit.co/download/rstudio-desktop/

9. R package, stringr (version 1.5.1), https://stringr.tidyverse.org/

10. R package, dplyr (version 1.1.4), https://dplyr.tidyverse.org/

11. R package, ggplot2 (version 3.5.1), https://ggplot2.tidyverse.org/

12. R package, ggstatsplot (version 0.12.3), https://indrajeetpatil.github.io/ggstatsplot/

13 R package, readxl (version 1.4.3), https://readxl.tidyverse.org/

14. Fluorescence spectral viewer tools, *https://www.fpbase.org/spectra/*

*Note: This link can be used by readers to explore spectral viewer tools to select fluorophores compatible with their microscope settings and their fluorescent transgenes.*

## Procedure

**Figure 1.**
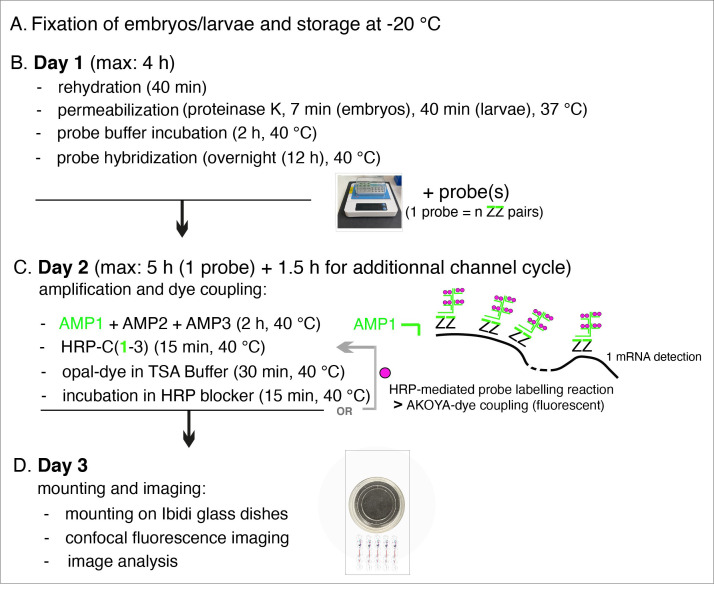
Summary of the main steps of the RNAscope procedure. AMP: signal AMPlifier (1, 2, or 3); HRP: HorseRadish peroxidase; HRP-C: HRP channel (1, 2, or 3); TSA: tyramide signal amplification; ZZ: pair of oligonucleotide probes [with target-specific binding sites covering regions of approximately 50 bases and mRNA target regions covering 300–1,000 nucleotides (with a pool of 6–20 ZZ pairs)]. OR + grey arrow indicates, on Day 2, the repeat of steps involved in additional fluorophore coupling using HRP activity in case of probe multiplexing using other channels (two times if two probes, three times if three probes) or interruption of the procedure until mounting of embryos/larvae on Day 3.

To get an overview of the procedure, which spans over three days, see Figure 1.

**A. Fixation of embryos and larvae, storage at -20 °C**

*Notes:*

*1. Embryo/larvae, dechorionated between 35 and 48 hpf with forceps, are fixed in 4% FA (15–20 maximum per tube), directly in 1.5 mL Eppendorf tubes (under a chemical hood). The tubes, with animals in methanol and kept at -20 °C until use, will then be easily handled for further use. For all washes, allow embryos/larvae to sediment by gravity; do not centrifuge.*

*2. Select under a fluorescence loupe the embryo/larvae with the best signal, as the transgene fluorescence will be used for quantitative analysis downstream and will not be amplified by immunofluorescence in the procedure described here (although the RNAscope protocol can be used in combination with immunochemistry).*

**Caution**: Depending on the fluorescent reporter used, FA and methanol fixation/storage at -20 °C can quench the fluorescence.

1. Fix anesthetized 48–50 hpf embryos or 5 dpf larvae in 4% FA diluted in PBST + tricaine 1/25, for 2–2.5 h, with gentle rocking on a horizontal rocking platform, at RT.

2. Wash twice with 1 mL of PBST, remove as much PBST as possible, and add 1.5 mL of 100% MeOH. Keep at -20 °C until use.

**B. Day 1: Rehydration, permeabilization, and probe hybridization**

1. Rehydration post-methanol fixation

*Note: Throughout the entire procedure, between each incubation and wash, embryos/larvae are sedimented by gravity. For 1 mL of volume, this step ranges from approximately 30 s at the beginning of the procedure to 1–2 min after starting incubations in probe diluent (PD) for larvae and can increase to 2–3 min for embryos (taping tubes with the finger can be done if embryos/larvae are sticking to the wall).*

a. Add 1 mL of 75% MeOH/mQH_2_O and incubate for 10 min on a horizontal rocking platform at RT. Allow embryos/larvae to sediment by gravity and remove supernatant.

b. Add 1 mL of 50% MeOH/mQH_2_O and incubate for 10 min on a horizontal rocking platform at RT. Allow embryos/larvae to sediment by gravity and remove supernatant.

c. Add 1 mL of 25% MeOH/mQH_2_O and incubate for 10 min on a horizontal rocking platform at RT. Allow embryos/larvae to sediment by gravity and remove supernatant.

d. Wash twice with 1 mL of PBST.

2. Deactivation of endogenous peroxidase activity

*Note: This step is necessary to reduce endogenous background.*

a. Let the embryos/larvae sediment and remove as much PBST as possible.

b. Add 75 µL/tube (three drops sequentially, taping the tubes with the finger after adding the last drop; each drop = 25 µL, approximately) of the H_2_O_2_ solution provided in the Multiplex Fluorescent Reagent kit v2. Incubate embryos/larvae for 10 min, on the rocking platform, at RT.

c. Let the embryos/larvae sediment and remove as much H_2_O_2_ solution as possible.

3. Permeabilization and protease digestion

a. Incubate embryos or larvae with 500 µL of proteinase K in PBST (1/2,000 from the glycerol stock at 20 mg/mL kept at -20 °C) for 7 min or 40 min, respectively, on a dry heating block, at 37 °C. For the 40 min incubation, in the case of larvae, shake the tubes gently every 10 min (by tapping the tubes with the finger).

**Figure 2.**
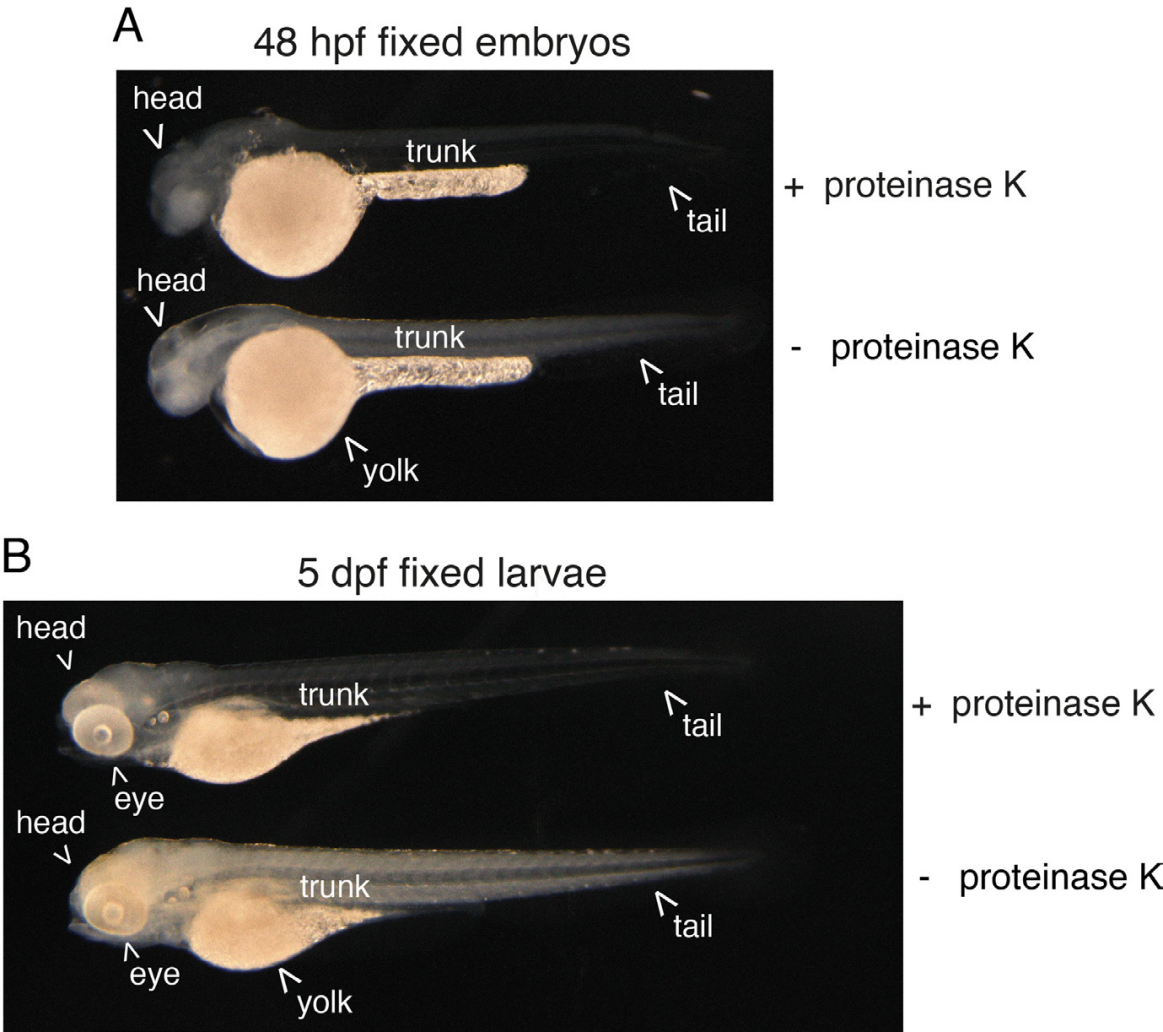
Efficiency of proteinase K digestion on the zebrafish embryo and larva. 48 hpf embryos (a) and 5 dpf larvae (b) were enzymatically digested or not (+ proteinase K, - proteinase K, respectively) after formaldehyde and methanol fixation. Note the effect of proteinase K treatment that is visualized by an increase in transparency of both the embryo and larva. In the embryo, the brain also appears more “milky,” which indicates the extreme limit of acceptable digestion.

**Critical**: Controlling the timing of enzymatic digestion is crucial for embryos; we recommend, depending on the batch of proteinase K, to control timing by looking at embryos under the binoculars. The head (brain region) of the embryos should turn “milky.” For both embryos and larvae, the body should become translucent but the yolk should not break off. See Figure 2 showing the expected digestion efficiency.

b. Sediment embryos/larvae by gravity and remove the permeabilization solution gently.

**Caution**: Remove the solution under the binoculars to avoid losing embryos/larvae.

c. Wash twice for 10 min in 1 mL of PBST with gentle rocking on the platform at RT (perform sedimentation steps on the bench and buffer removal as described in step B3b.

4. Probe(s) hybridization

*Notes:*

**Figure 3.**
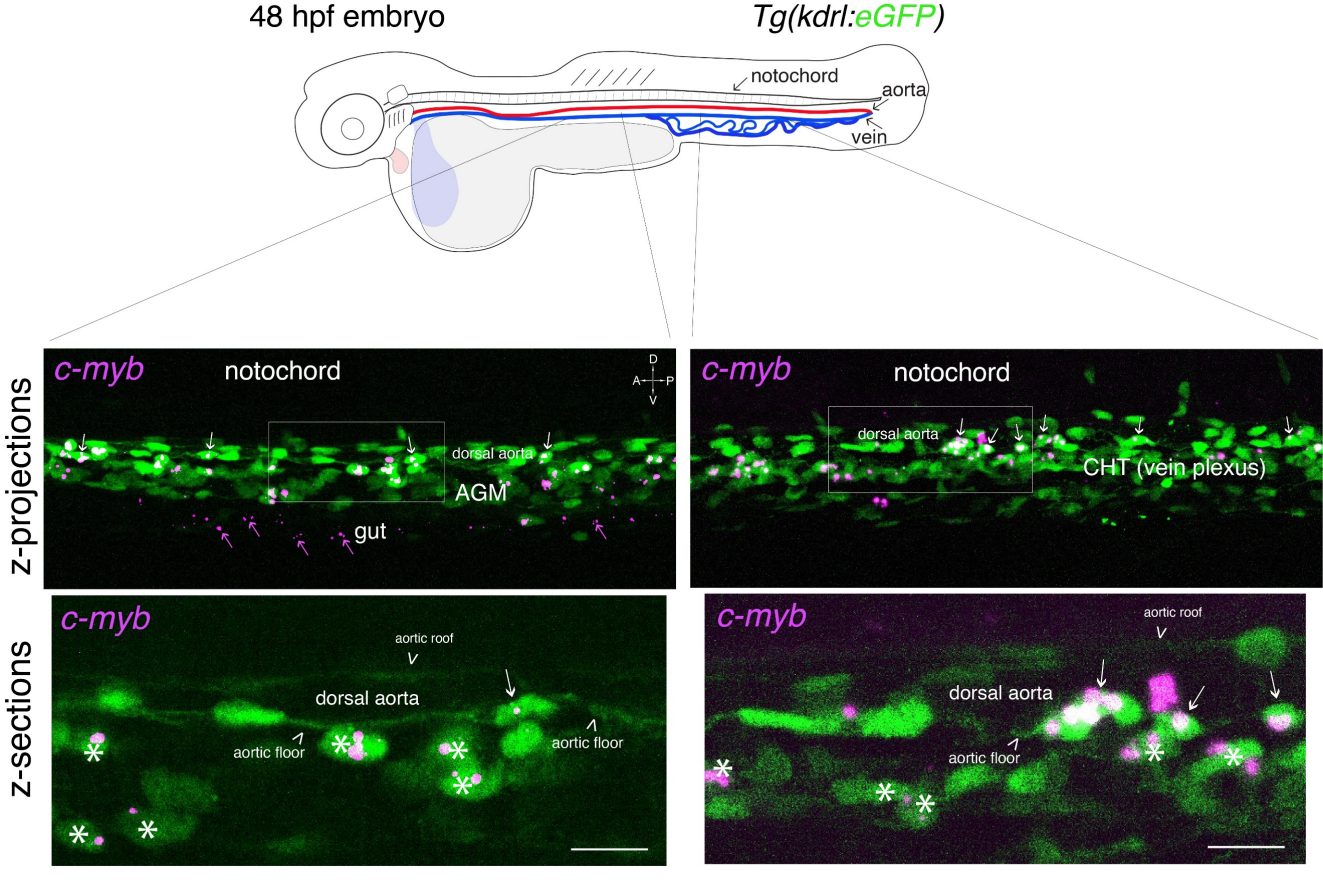
Whole-mount in situ analysis of *cmyb* mRNA expression in a 48 hpf embryo . Top panel: schematic representation of a 48 hpf embryo (reproduced with modifications from [16]); red line = aorta (from which hematopoietic stem cell precursors emerge); blue line = vein (showing particularly the vein plexus, constituting the CHT). The embryo used expresses eGFP under the control of the vascular *kdrl* promotor [*Tg(kdrl:*eGFP) fish line]. Bottom panels: spinning disk confocal images obtained either in the trunk region (left: AGM) or in the tail region (right: CHT). Images shown were obtained either from maximal z-projections of z-stacks or from single z-sections with 3× magnification of regions in white boxes. Green channel: vascular cells expressing soluble eGFP [aortic and vein endothelial cells, including cells of the hemogenic endothelium (HE; white arrows) in the trunk and tail regions] as well as newly born HSPCs (white asterisks). Magenta: RNAscope spots indicating expression of *cmyb* mRNAs in the HE and in HSPCs. Note the presence of RNAscope spots in the trunk (top left image) and in the gut region (magenta arrows) that indicate the potential expression of *cmyb* in more differentiated patrolling immune cells. AGM: aorta gonad mesonephros; CHT: caudal hematopoietic tissue; HSPCs: hematopoietic stem and progenitor cells. Scale bars = 20 μm.

**Figure 4.**
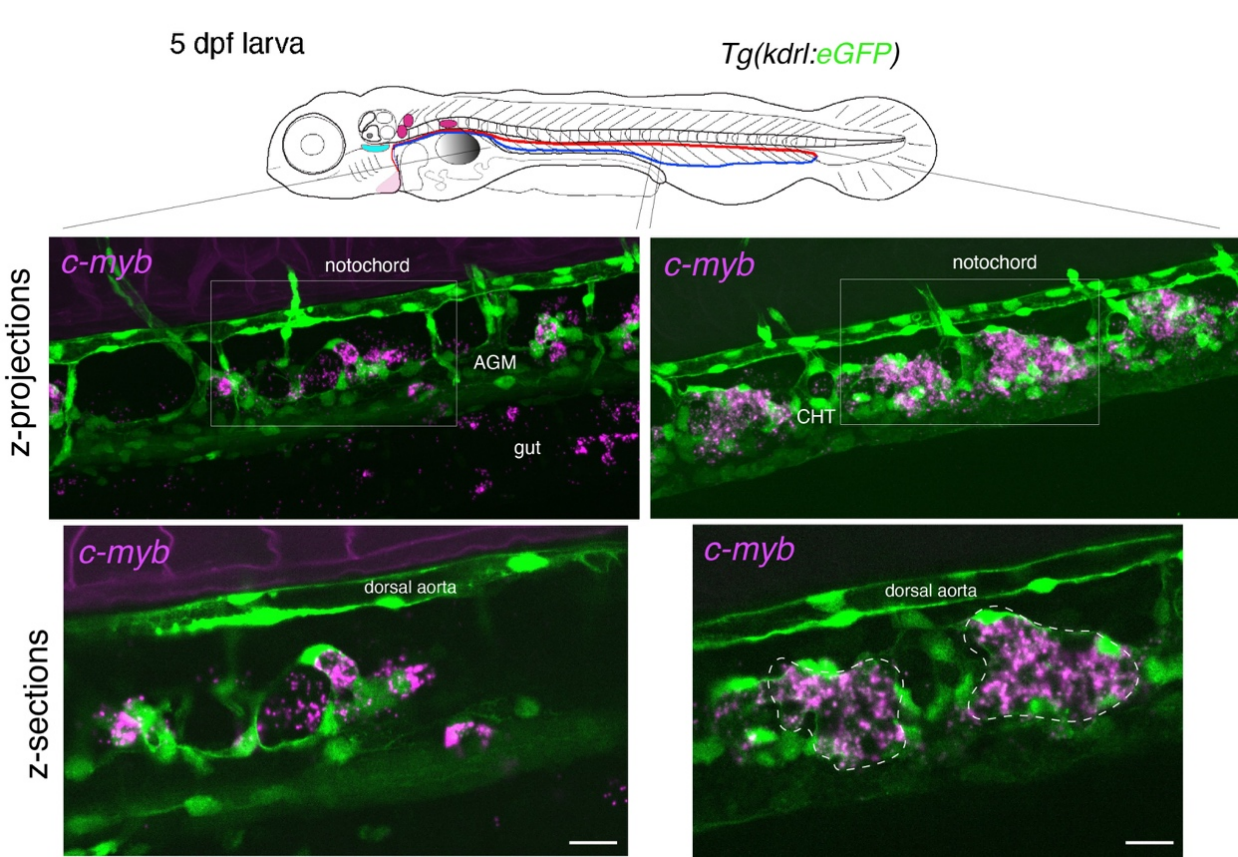
Whole-mount in situ analysis of *cmyb* mRNA expression in a 5 dpf larva . Top panel: schematic representation of a 5 dpf larva (reproduced with modifications from [16]); red line = aorta; blue line = vein. The larva used expresses eGFP under the control of the vascular *kdrl* promotor [*Tg(kdrl:*eGFP) fish line]. Bottom panels: spinning disk confocal images obtained either in the trunk region (left: AGM) or in the tail region (right: CHT). Images shown were obtained either from maximal z-projections of z-stacks or from single z-sections with 1.5× magnification of regions in white boxes. Green channel: vascular cells expressing soluble eGFP (aortic and veinous endothelial cells in the trunk and tail regions). Magenta: RNAscope spots indicating expression of *cmyb* mRNAs in HSPCs. Note that RNAscope signals cannot be superposed to individual cells because HSPCs are no longer expressing eGFP driven by the vascular *kdrl* promoter, as is the case in 48 hpf embryos (owing to distant timing from their emergence, half-life of eGFP, and division cycles diluting the fluorescent protein). Note the structural evolution of the vein niche, notably in the CHT, in comparison with the 48 hpf embryo shown in Figure 3, and the relatively regular positioning of hematopoietic clusters (delimited by dashed lines in the bottom right image). AGM: aorta gonad mesonephros; CHT: caudal hematopoietic tissue; HSPCs: hematopoietic stem and progenitor cells. Scale bars = 20 μm.

**Figure 5.**
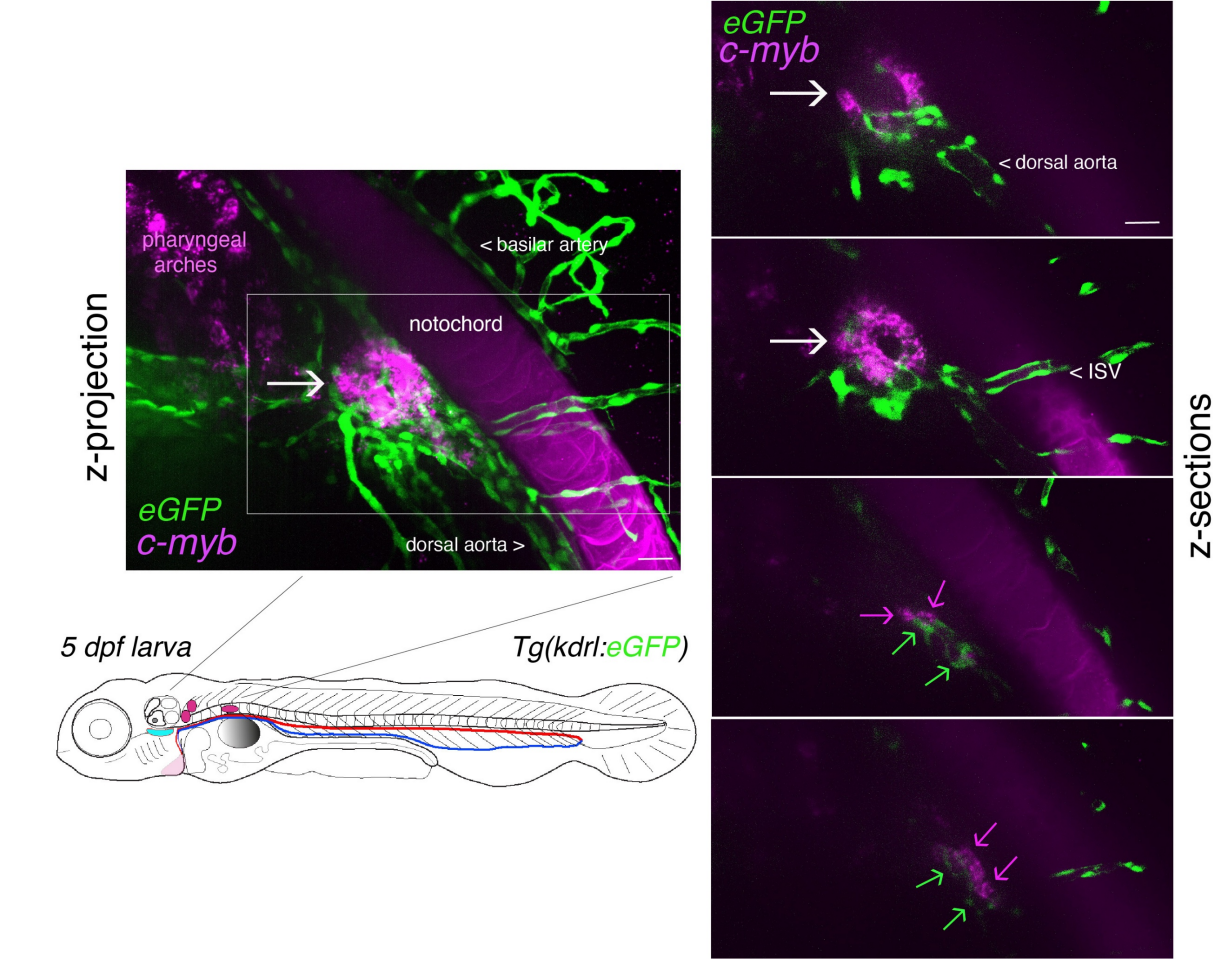
Whole-mount in situ analysis of RNAscope signals deep in the pronephros region of 5 dpf larvae using the *cmyb* probe. Spinning disk confocal images obtained with a 5 dpf larva [*Tg(kdrl:eGFP*) fish line] expressing eGFP in endothelial cells under the control of the vascular *kdrl* promotor. Images show the pronephros region with a maximal z-projection (left) of a z-stack encompassing a total of 240 sections, each interspaced by 0.5 μm, and z-sections (right: images of sections 52/240, 86/240, 186/240, and 222/240 from top to bottom, respectively). The *cmyb* RNAscope signals (magenta) correspond to hematopoietic stem and progenitor cells (HSPCs) accumulated in the peri-glomerular region (white arrow), surrounded by vessels. Note the more distant localization of *cmyb* signals (magenta arrows, hypothetically in HSPCs) along vessels (green arrows). This unveils the potential complexity of the pronephros niche. ISV = intersegmental vessel. This z-stack is also visualized in Video 1 and reconstituted in 3D using Imaris in Video 2. Note also the nonspecific capture of the dye in the notochord, which facilitated the localization of the glomerulus. Scale bars = 20 μm.

**Figure 6.**
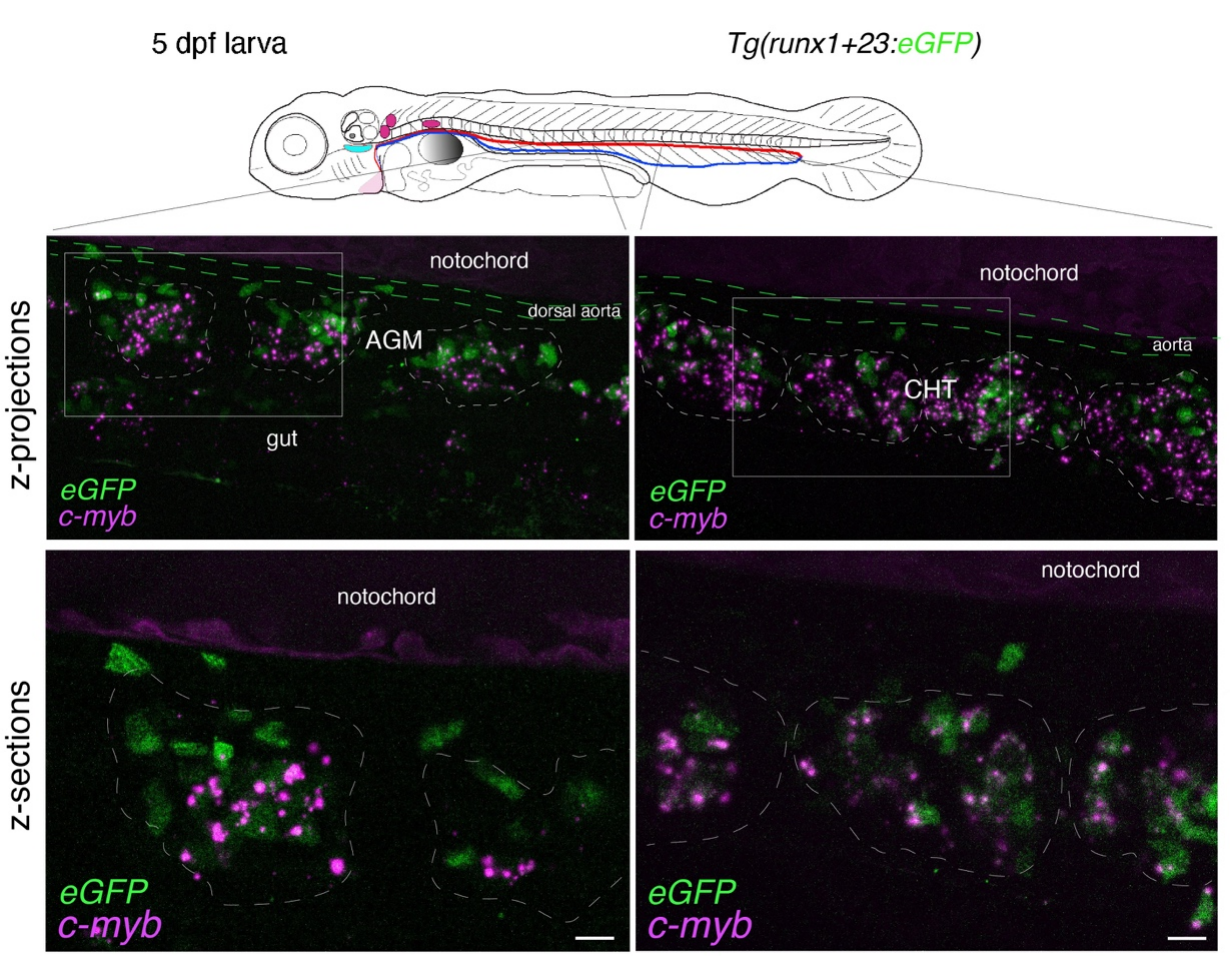
Whole-mount in situ analysis of *cmyb* mRNA colocalization with HSPCs at 5 dpf. Spinning disk confocal images obtained with a 5 dpf larva [*Tg(runx1+23:eGFP*) fish line] expressing eGFP in HSPCs under the control of the *runx1+23* enhancer. Panels are constituted from images obtained either in the trunk region (left: AGM) or in the tail (right: CHT), either from maximum z-projections of z-stacks or from single z-sections with 2× magnification of regions in white boxes, highlighting hematopoietic clusters (dashed lines). RNAscope signals (magenta) colocalize in the majority with HSPCs (see also Figure 7 and Video 3 for 3D reconstitution). AGM: aorta gonad mesonephros; CHT: caudal hematopoietic tissue; HSPCs: hematopoietic stem and progenitor cells. Scale bars = 20 μm.

**Figure 7.**
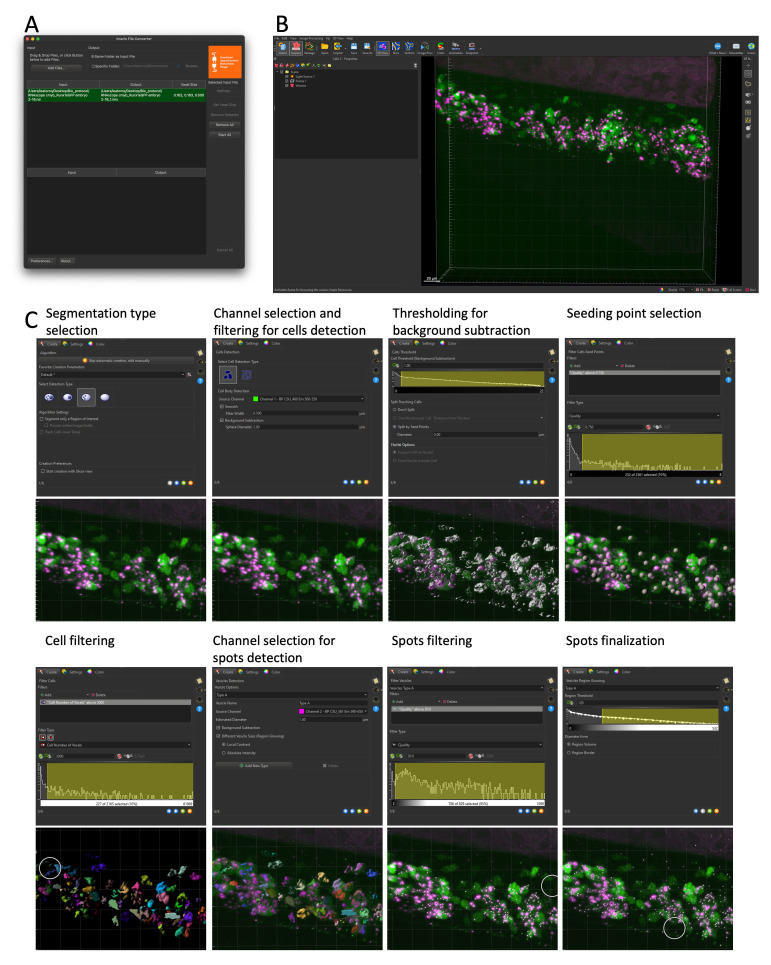
Cell and RNAscope segmentation workflow with Imaris. All data shown are extracted from a single caudal hematopoietic tissue (CHT) z-stack showing the expression of *cmyb* (RNAscope, spots in magenta) in hematopoietic cells expressing eGFP under the control of the *runx1+23* enhancer (the same 5 dpf larva and images as in Figure 6). (A) File conversion with Imaris File Converter. (B) Imaris 3D visualization in surpass mode. (C) Step-by-step workflow of cell segmentation, as detailed in Data Analysis, Section B. *3D cells and RNAscope signal segmentation with Imaris – Cell Biologist package*.

*1. The protocol beneath is for incubation with one probe, which is illustrated in Figures 3–7 beneath. Up to three probes can be co-incubated at the hybridization step if they are in the C1, C2, and C3 channels, respectively. Since probes in the C1 channel are delivered diluted in probe diluent (PD), the probe solution can be applied directly to the samples. If the probes are in the C2 or C3 channels, they should be diluted 1/50 in PD before addition to the samples.*

*2. Control probe(s), ideally in each one of the channels that are used for assays with specific probes in a given channel, should be assayed in parallel to appreciate background (which can also vary with specific organs).*

*3. For our procedure, we use a heating block with a cap (see the reference in the Equipment list and Figure 1); although never tested, an alternative would be to use a moisture-controlled oven, particularly for the overnight incubation with the probe(s).*

a. Incubate embryos/larvae with 100 µL of PD for at least 2 h, on the heating block, at 40 °C.

b. Remove as much PD as possible and replace with 50–100 µL/tube of the probe (or a mixture of several probes).

c. Incubate overnight in the heating block at 40 °C.

**Caution**: Since the heating block is not agitating (to prevent damaging embryos/larvae), mix gently the content of the tube(s) a couple of times before the overnight incubation by gently tapping the tubes with the finger.

**Video 1.**
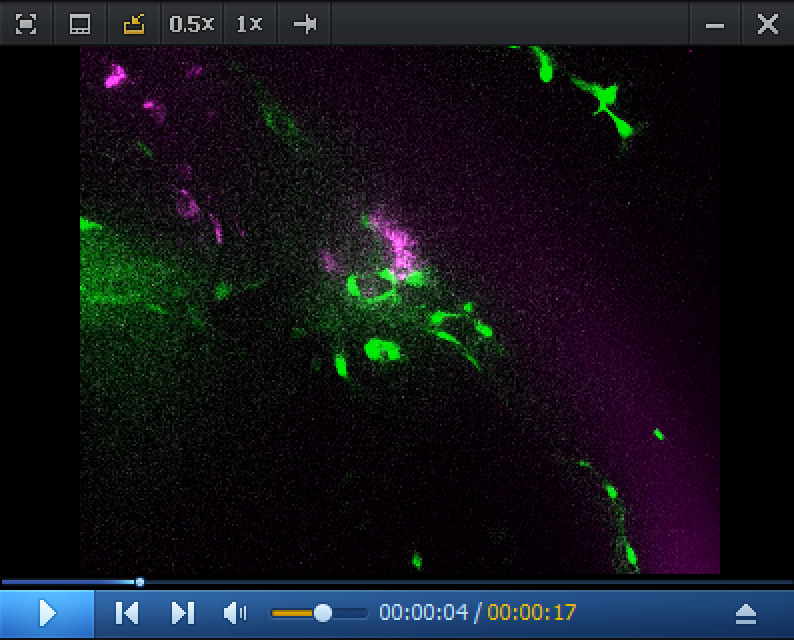
RNAscope in situ *cmyb* expression analysis in the pronephros region at 5 dpf. Spinning disk confocal images (z-stack) obtained from a 5 dpf *Tg(kdrl:eGFP*) larva. The eGFP is expressed in endothelial cells under the control of the vascular *kdrl* promotor. Z-stack shows the pronephros region; consecutive confocal z-sections are 0.5 μm interspaced. The *cmyb* RNAscope signals (magenta) are localized in hematopoietic stem and progenitor cells (HSPCs) accumulated in the peri-glomerular region, surrounded by vessels. Unique z-sections (52/240, 86/240, 186/240, and 222/240) and maximum z-projection are also shown in Figure 5. This z-stack is also reconstituted in 3D in Video 2.

**Video 2.**
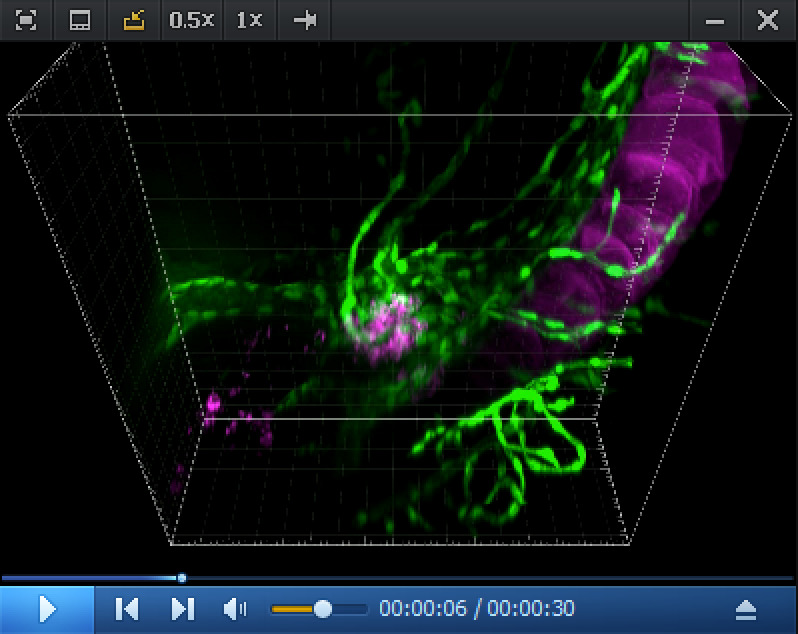
Imaris 3D reconstitution of RNAscope in situ *cmyb* expression analysis in the pronephros region at 5 dpf. 3D rendering (reconstituted with the Imaris software) obtained from a spinning disk confocal image (z-stack) obtained from a 5 dpf *Tg(kdrl:eGFP*) larva. The eGFP (green) under the control of the *kdrl* vascular promoter is expressed in endothelial cells. Z-stack shows the pronephros region. The *cmyb* RNAscope signals (magenta) are localized in hematopoietic stem and progenitor cells (HSPCs) accumulated in the peri-glomerular region, surrounded by vessels. Unique z-sections and maximum z-projection are also shown in Figure 5, and the complete z-stack is shown in Video 1.

**Video 3.**
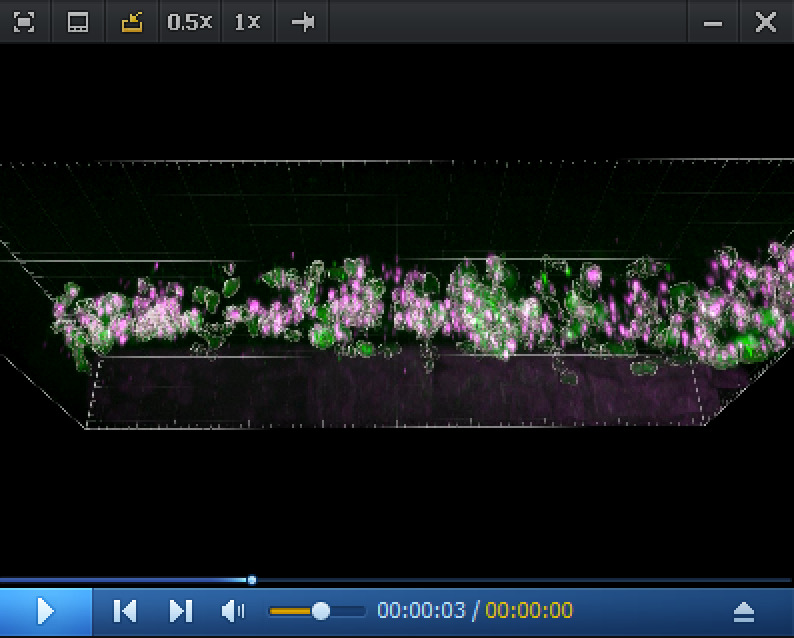
Imaris 3D rendering and colocalization of RNAscope spots and hematopoietic stem and progenitor cells (HSPCs) in the CHT region. 3D reconstitution generated from a spinning disk confocal z-stack obtained from a 5 dpf *Tg(runx1+23:eGFP)* larva. eGFP (green) under the control of the *runx1+23* enhancer is expressed in HSPCs. RNAscope signals (magenta) colocalize in the majority with HSPCs (see also Figure 6 for maximum z-projections and single z-sections and Figure 7 for 3D reconstitution and cell segmentation steps).

**C. Day 2: Amplification and fluorescence reaction**

*Note: The Multiplex v2 kit allows performing HRP-based tyramide signal amplification (TSA) using fluorescent dyes applicable to up to three probes concomitantly (3-plex assay). Here, we show confocal fluorescence images [Figure 3 (n = 1, 48 hpf embryo, trunk and tail regions), Figures 4 and 5 (n = 1, 5 dpf larva, trunk, tail, and pronephros regions), and Figure 6 (n = 1, 5 dpf larva, trunk and tail regions)] obtained using one probe only (single plex assay), with amplification reactions revealing a 570 nm fluorescent dye (OPAL-570). Assays were also performed with two probes concomitantly but with difficulties regarding the maintenance of the morphology of embryos/larvae due to additional steps of incubation/washes at the HRP reaction step. In this specific case, we revealed the two probes using OPAL-570 and OPAL-690. OPAL-480 (see Reagents) can be used as an alternative to one of the dyes, depending on the strategy followed for the experiment and imaging setup (here, the use of eGFP transgenic fish line is not compatible with this dye).*

1. Amplification and dye coupling

*Note: Prewarm (for 10 min, on the surface of a heating block, at 30 °C) the concentrate 50× WB (provided with the kit) before shaking the bottle and diluting 1/50. Before each step with specific reagents provided in the kit [AMP(1–3) reagents, FL v2 HRP-C(1–3) reagents, TSA buffer], place aliquots of the required volume (depending on the number of samples) on the heating block for 5 min (prewarming at 40 °C). For replacement of all reagents and WB, sediment embryos/larvae by gravity on the bench and remove media under the binoculars. From time to time, during incubations at 40 °C on the heating block, shake very gently by tapping tubes with the finger.*

a. Wash each sample twice with 1 mL of WB for 10 min and with gentle rotative agitation on the rocking platform at RT.

**Critical**: Overnight incubation with probes in PD and following washes in WB trigger the clearing of embryos/larvae; all steps of reagents and WB removal after incubations need to be performed under binoculars to avoid losing material. This recommendation is important because the proteinase K digestion has already triggered the translucence of material, which is critical particularly for handling 48 hpf embryos. In addition, embryos/larvae will lose density and will take longer to sediment by gravity; do not centrifuge because embryos/larvae will curve, which will make mounting and imaging more difficult.

b. Remove WB and add 50 µL of AMP1. Incubate for **30 min at 40 °C** on the heating block. Add 1 mL of WB and sediment embryo/larvae by gravity. Remove medium and wash twice with 1 mL of WB for 10 min and with gentle rotative agitation on the rocking platform at RT.

c. Remove WB and add 50 µL of AMP2. Incubate for **30 min at 40 °C** on the heating block. Add 1 mL of WB and sediment embryo/larvae by gravity. Remove medium and wash twice with 1 mL of WB for 10 min and with gentle rotative agitation on the rocking platform at RT.

d. Remove WB and add 50 µL of AMP3. Incubate for **15 min at 40 °C** on the heating block. Add 1 mL of WB and sediment embryo/larvae by gravity. Remove medium and wash twice with 1 mL of WB for 10 min and with gentle rotative agitation on the rocking platform at RT.

*Note: Even if only one probe is used (for example, in Figures 3–7), incubations with the AMP1, AMP2, and AMP3 solutions are required, particularly for comparative studies using probes in different channels, which will ensure regularity in background levels.*

e. Remove WB and, for each sample, add 50 µL of RNAscope Multiplex FL v2 HRP-C(1–3) reagent. Incubate for **15 min at 40 °C** on the heating block. Add 1 mL of WB and sediment embryos/larvae by gravity. Remove medium and wash twice with 1 mL of WB for 10 min and with gentle rotative agitation on the rocking platform at RT.

*Note: In Figures 3–7, we used the cmyb-C3 probe. Hence, we have performed the reaction with the RNAscope Multiplex FL v2 HRP-C3 reagent.*

f. Remove WB and add, for each sample, 100 µL of TSA buffer. Incubate for 10 min on the heating block at 40 °C.

g. Remove the 100 µL of TSA buffer and add 500 µL of the diluted (1/500) OPAL-570 dye (or any other appropriate OPAL dye depending on the required fluorescence wavelength). Incubate on the heating block for **30 min at 40 °C**. Sediment embryos/larvae by gravity. Remove medium and wash twice with 1 mL of WB for 10 min and with gentle rotative agitation on the rocking platform at RT.

h. Remove WB and, for each sample, add 50 µL of the HRP blocker solution. Incubate on the heating block for **15 min at 40 °C**. Add 1 mL of WB and sediment embryos/larvae by gravity. Remove medium and wash twice with 1 mL of WB for 10 min and with gentle rotative agitation on the rocking platform at RT.

*Note: If the reaction is done with one channel only, the protocol can be stopped here (as performed for results shown in Figures 3–7). If required, a DAPI staining of nuclei can be performed. In any case, at the end, keep the embryos/larvae in PBST until mounting. If more than one probe was hybridized, continue the protocol by returning to step C1e; incubate with HRP coupled to another channel [e.g., HRP-C2 if the first reaction was performed with HRP-C1 and if the second probe was synthesized in the C2 channel (or HRP-C3 if synthetized in the C3 channel)]. In the latter case(s), the DAPI reaction can be performed at the end of the protocol (and/or continuing with immunofluorescence if required).*

**D. Day 3: Mounting and imaging**

*Note: Mounting can also be performed at the end of Day 2. Ideally, imaging should be performed on Day 3 but imaging on day 4 is also possible without significant degradation of the biological material (even without 0.02% NaN_3_ or any other preservative, with mounted samples kept in a cold room at 4 °C).*

1. Mounting

**Figure 8.**
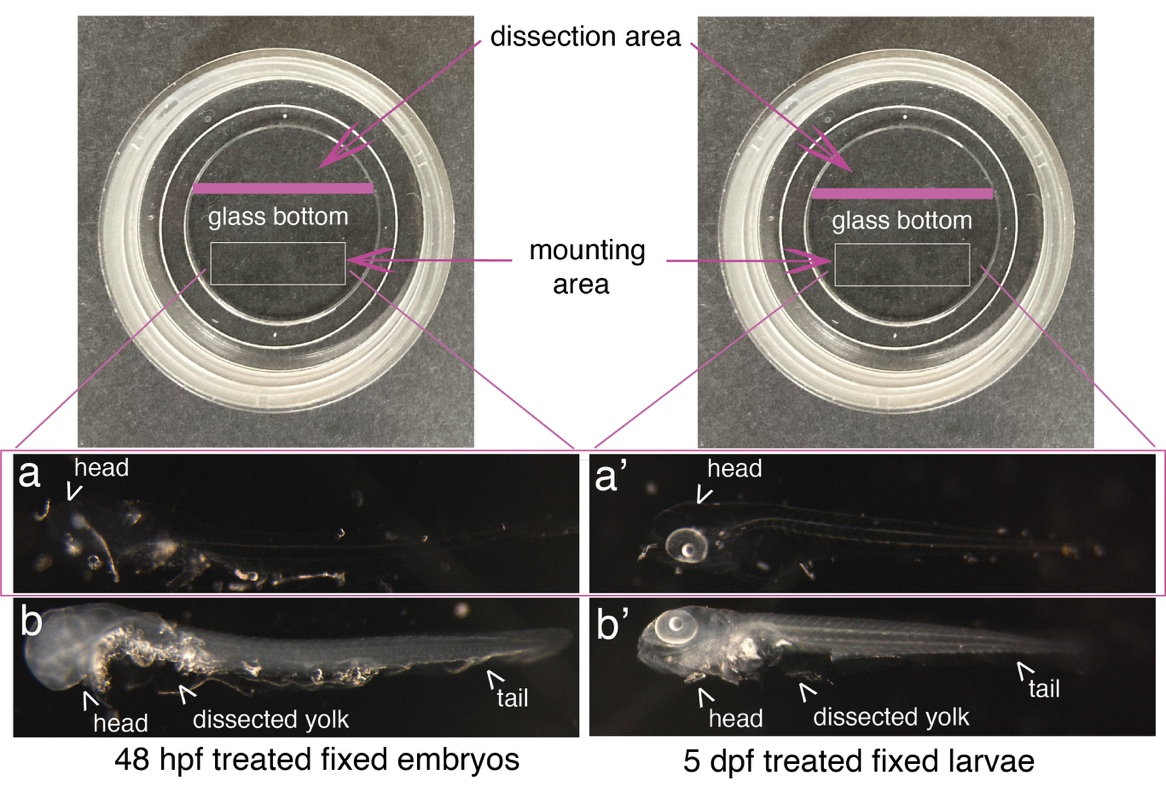
Mounting of embryos and larvae after RNAscope reagents treatment. Top images show Ibidi dishes, with the recommended dissection area on the top and the agarose mounting area at the bottom (white rectangles). 48 hpf embryos (a, b) and 5 dpf larvae (a’, b’) were either (a, a’) or not (b, b’) treated with proteinase K and all of them with RNAscope reagents. Images show that the whole procedure leads to virtually total transparency of the specimen, which reaches a maximum for embryos [becoming barely visible as in (a)]. This transparency requires controlling all steps of reagent/buffer removal under the binoculars. Yolks were dissected, as well as the eyes facing the glass bottom of the dish to ensure flatness.

**Critical:** The mounting step is critical because embryos/larvae are transparent, sticky, and very fragile. Perform all steps under the binoculars. To prevent losing embryos/larvae, mounting is processed in the same Ibidi dish as the dissection of yolk and protruding eyes (removing eyes with 25G needles, particularly the one facing the glass bottom of the Ibidi dish, will ensure flatness of the mounting). More specifically, the eye facing the glass needs to be removed if imaging anterior niches (the thymus, the pronephros, or any other organ in the anterior region). Any dissection is performed on a restricted area of the glass (e.g., the upper part; see Figure 8).

a. Remove WB (in case the mounting is performed directly after amplification and dye coupling) and wash twice for 10 min in PBST with gentle rotative agitation on the rocking platform at RT.

b. Transfer up to 6–7 embryos/larvae from the Eppendorf tube to the Ibidi dish (maximum volume of 1 mL; if the volume of animals and PBST is below 1 mL, complement up to 1 mL with PBST; this volume will cover the surface of the glass bottom).

c. Carefully remove the eyes and protruding tissues that may hamper the flatness of the mounting (with 25G needles).

d. Remove medium and debris and add 1 mL of PBST.

e. Move the embryos/larvae with a slightly trimmed Eppendorf microloader to the middle/bottom of the dish; the fact that they stick to the glass will help to maintain them longitudinally on the lateral side (Figure 8).

f. Slowly add 1 mL of 2% agarose (melted by warming up at 85 °C on the heating block; see the note below) while mixing very gently to limit perturbations to the embryos/larvae alignment. If necessary, reorient them longitudinally. Wait until the agarose is solidified before moving the dishes.

*Note: Prepare the 2% low melting agarose in 1*× *PBS. Typically, 30 mL of 1*× *PBS is added to a 50 mL Falcon tube. Warm up the solution in a microwave until nearly reaching the boiling point with the tube uncapped (wear protective glasses and gloves). Add the agarose powder slowly and stepwise, close the cap of the Falcon tube, and shake vigorously. Repeat warming up, adding agarose stepwise and mixing until the entire solution is nearly entirely dissolved. Complete the volume to 50 mL, warm up, close the tube, and shake. Aliquot in 2 mL Eppendorf tubes. When needed for mounting, warm up the Eppendorf tubes containing 2% agarose to 85 °C until complete melting. Transfer 1 mL of the agarose in a new 2 mL Eppendorf tube and immediately perform 3–4 ups and downs to homogenize and cool the temperature down before mounting by slowly mixing the 2% agarose with 1 mL of PBST containing embryos/larvae properly dissected in the Ibidi dish. The appropriate temperature when initiating mounting can be maintained by slowly releasing the 2% agarose from the 1 mL tip at the external plastic margin of the dissection area (see Figure 8); no vapor should be seen on the margin (which, before the addition of the 2% agarose, should remain dry while the 1 mL PBST is contained in the area delimited by the glass bottom of the Ibidi dish).*

**Pause point**: Imaging can be performed on the day following the mounting. In this case, keep the dishes protected from light in a cold room.

2. Confocal imaging and interpretation of RNAscope information

Confocal imaging is specific to each setup, depending on available equipment; for an example of imaging conditions of 48 hpf embryos treated for RNAscope, see [8]. Hence, this part of our protocol is not aimed at describing image acquisition but at guiding the reader to critically analyze the outcome of the RNAscope procedure, here applied to zebrafish 48 hpf embryos/5 dpf larvae but that can be extended to other stages or organisms. This section is then organized as a series of points (Figures 3–6, Videos 1 and 2) to guide throughout specific and relevant points of RNAscope analysis after image acquisition, including the characteristics of the RNAscope signals with the multiplex assay (notes 1 and 2), the specific case of in-depth signals (note 3), and the choice of appropriate biological material and configuration to quantify RNAscope signals per cell type (note 4, linking to the Data analysis section).

a. To obtain information throughout the entire organ/region of the embryo and at the cellular resolution, a series of z-sections needs to be acquired. In Figure 3, a 48 hpf embryo expressing the *eGFP* controlled by the vascular *kdrl* promoter [*Tg(kdrl:eGFP*)] was imaged through a series of z-sections in the trunk and in the tail regions (observed with a 40× objective). Images reveal the typical RNAscope spotty signals obtained with the multiplex assay.

To quantify the number of spots and avoid saturation, all fluorescent confocal images were obtained in the linear range of the camera and analyzed without modification of the brightness and contrast. Importantly, quantitative analyses using RNAscope can only be applied to spot numbers and not intensity (in theory, 1 spot = 1 mRNA; for more details the Data analysis section). Larger and more intense spots are assumed to correspond to signals produced by several mRNAs accumulating in an area below the resolution of the optic microscope. Regarding spot fluorescence intensity, their respective minimal and maximal intensities give an estimation of the dynamic range of signals in comparison to the background. Typically, for the results shown in Figure 3, RNAscope signals are, for the highest intensity spots, 53 times higher than the background; for the lowest intensity spots, signals are 3.3 times higher than the background.

b. For 5 dpf larvae, in the trunk and in the tail regions, the RNAscope spots are detected with high density, mostly because hematopoietic cells are organized into clusters (see Figure 4). The conditions of image acquisition were the same as for Figures 3–7; raw images, without modification of brightness and contrast, reveal individual spots (relatively well individualized in the z-sections shown in Figure 4, in which, however, image brightness and contrast was modified from the raw images for visibility). For a comparison with the signals analyzed in Figure 3, the fluorescence intensity is 98 times higher than the background for the highest intensity spots and 7.6 times higher for the lowest signals. To evaluate, in the 5 dpf larva, the association of RNAscope spots with individual hematopoietic cells (using the cmyb-C3 probe), see Figure 6.

c. In the pronephros region of 5 dpf larvae, in addition to the comments made for Figure 4 in point 2 above, imaging the region of interest in depth is a major issue. The peri-glomerular area is localized beneath the notochord, deep in the anterior region (see Figure 5 and the z-projection image on the left), hence decreasing image resolution and signal intensity as well as increasing diffusion of the fluorescence signal and overall background. In these conditions, using deep red dyes is not recommended (such as for example the OPAL-690 dye). One advantage is the capture, by the notochord, of the fluorescent dye that is useful to localize the peri-glomerular region as well as the more distant pronephric tubules and vascular structures around which HSPCs are niching (see Figure 5, Video 1, produced by assembling the individual slices of the z-stack, and Video 2, obtained by reconstituting the z-stack in 3D using the Imaris software).

d. In 5 dpf larvae, the RNAscope signal can be superposed to hematopoietic cells when using a transgenic fish line that expresses a fluorescent reporter driven by a hematopoietic promoter. For example, we used here a *Tg(runx1+23:eGFP)* line that allows visualizing HSPCs (mainly organized in clusters), expressing cytosolic eGFP as well as *cmyb* mRNAs (Figure 6). The RNAscope spots can then be colocalized with HSPCs expressing eGFP by 3D reconstitution of the z-stack using the Imaris software (see Figure 7).

## Data analysis

**A. Image analysis overview**

Images acquired with the confocal microscope are multichannel z-stacks that can be analyzed with open-source image analysis software [including but not limited to ImageJ (FIJI), Icy, and Napari [17–19]] or with proprietary software such as Imaris. Here, the foundation of the quantitative analysis lies in the segmentation of cellular volumes (labeled, in our case, by eGFP transgene fluorescence) as well as the segmentation of RNAscope spots and the possibility of detecting which cells contain spots. On more general grounds and in different biological contexts, analyses can lead to a diversity of quantitative results, such as (i) the total number of positive cells, (ii) the repartition of positive cells within an organ or—the case in our current studies—in embryos/larvae, (iii) the variation of percentage of positive cells in interfering conditions, and (iv) co-expression analyses in multiplex RNAscope assay.

The quantification of the number and size of spots per cell is possible but should be done carefully as confounding factors [the variation of the TSA amplification step duration between multiple tubes or across conditions, the number of ZZ-pairs used to detect each gene, and the localization of mRNA molecules within the cell (homogeneously dispersed or clustered together)] might bias the interpretation of the analysis by modifying the intensity and size of RNAscope spots.

Importantly and to avoid misinterpretation, image analysis should be performed with software and workflows designed for 3D analysis. For instance, working with 2D projections (such as maximum intensity projections; see, for example, the z-projections in Figures 3–6) of z-stacks will lead to the observation of falsely positive cells, when RNAscope signal overlaps with transgene fluorescence in the x- and y-axis but not the z-axis.

To perform qualitative analysis and visualize images, Napari and Imaris Viewer are free options that can allow exploring data with fast 3D reconstitution (3D rendering is the default visualization mode), while ImageJ (*Image* → *Stack* → *3D project*…) and Icy (*3D VTK*) propose slower and less manipulable renderings, with a noticeable reduction of the displayed image quality. All allow to hide or show specific channels, zoom on the data, and change the color and intensity display, but 360° rotation of the 3D stack is not supported in ImageJ.

For the creation of a quantitative analysis workflow, we use the Imaris software (version 10.1.0) with the complete workflow detailed below (see also https://imaris.oxinst.com/tutorials for various Imaris tutorials). Because Imaris requires a license, we will briefly mention two free alternative software and possible workflows (ImageJ and Napari).

1. ImageJ

With ImageJ, the 3D ImageJ Suite and MorpholibJ suite [20,21] propose solutions to perform segmentation of cells and spots (*Analyse* → *3D object counter, Plugins* → *3D* → *3D spot segmentation*). To facilitate segmentation, pre-processing (filtering: *Plugins* → *3D* → *3D fast filters*) can be used. Once segmented objects have been obtained, segmentation data can be modified (merge, split, remove) and saved using the 3D object manager (*Plugins* → *3D* → *3D manager*). With the same manager, .csv files containing information regarding segmented objects can be exported, including fluorescence intensity, size, position, or geometrical information. The segmentation workflow can be made into a macro (*Plugins* → *macro* → *record)*, allowing you to run your different steps, recording it as a script, and saving it (see https://imagej.net/scripting/macro for detailed use of the macro tools), and reusing it automatically on multiple files (*Plugins* → *macro* → *run*). The main limitation of the ImageJ suite is the absence of object-object interaction analysis: spots and cells can be segmented, but one cannot automatically get information on the position of spots relative to cells. One can deduce if a segmented cell is positive, based on the intensity of fluorescence in the RNAscope channel, but the number or size of RNAscope spots within a cell cannot be extracted. A script (macro) is available as an example at https://doi.org/10.5281/zenodo.14936123.

2. Napari

With Napari, the Zelda plugin [22] provides a single package that allows for pre-processing, segmentation, relation analysis between multiple object types, and generation of data tables and plots from extracted information. Napari is a relatively recent Python interface for image visualization and analysis; as such, it requires minimal knowledge of coding to use the majority of its plugins or to create a custom pipeline. One of the advantages of Napari is its versatility and the constant creation and upgrading of the available packages by the user community. The Zelda plugin provides a graphical interface to facilitate its utilization. One of the shortfalls of the Zelda package compared to the Imaris software is the reduced ability to interact with the objects after segmentation (manual curating: split, merge, suppression, classification, sorting). Moreover, Zelda allows for the concomitant analysis and segmentation of only two object types, and therefore simultaneous analysis of multiple RNAscope targets in multiplex experiments is not supported.

**B. 3D cells and RNAscope signal segmentation with Imaris, Cell Biologist package**

1. Convert the acquired individual confocal image files into proprietary .ims files using the Imaris file converter software (*Add files*). Specify, if needed, the voxel size (*Set Voxel Size*) and convert one or multiple files simultaneously (*Start All*) (Figure 7A). New .ims files are automatically generated in the same folder as input files.

2. Open the .ims file and select the *3D View* in the Surpass mode to generate a 3D rendering of the z-stack (Figure 7B). The channel color display can be changed as well as the displayed intensity (*Edit* → *Show Display Adjustment*).

3. Depending on your needs, Imaris offers multiple segmentation tools (*Spots, Surface, Cell, Filaments*). Here, we used the *Cell* tool, a combination of the *Spots* and *Surface* tools, that allows for the simultaneous segmentation of cells and objects (nuclei, vesicles) within the cells. Once selected, one can choose to use either the creation wizard with modifiable pre-defined parameters or manually parameter all steps. Of notice, the manual configuration can be used to import objects already segmented (such as the already defined *Spots* and *Surface* objects to generate integrated *Cell* objects). Here, we used the creation wizard and will detail each step (Figure 7C).

a. First step. Here, one can choose to load saved parameters from previous experiments, by clicking the tool button next to the *Favorite Creation Parameters*, and loading from the computer a .icpx file. Similarly, at the end of segmentation, one can export parameters for future experiments (*Store Parameters for batch*). Here, we went through all eight steps to generate *Cell* objects. One can move to the next step by clicking the blue arrow at the bottom of the window or fast forward to the end of the wizard by clicking on the double green arrow (after loading pre-defined parameters, for example).

**Caution:** Parameters can be reused for similar experiments (same transgenic background with identical pattern of fluorescence, same RNAscope probe), but must be re-evaluated for each new experimental setup; thresholding for background intensity and cell or spot size, for example, will differ in different transgenic lines and with different expression of the genes investigated.

First, for our image analyzed here (Figure 7C), we selected amongst the four options the one corresponding to our data (A *Surface* object containing multiple *Spots* objects). Since our image is relatively small, we performed the segmentation of the whole image straightaway; for bigger images, the *Segment only a region of interest* option allows running the wizard in a small ROI and only after expanding the segmentation to the whole image, to speed up the calculations during the configuration steps.

b. Configure cell type: Chose whether the fluorescence signal is cytoplasmic (our case) or restricted to the membrane (such as a ZO1-eGFP signal), select the channel used for the cell segmentation, and apply pre-processing filters (*Smooth*: gaussian filter, *Background subtraction*).

c. Set the *Cell Threshold (Background Subtraction)*: The threshold proposed by the wizard, corresponding to the limit between signal and background noise, can be manually modified. Here, if cells are tightly packed and touching each other, manual splitting can be performed, using the *Split Touching Cells* function. In our case, because we lacked a nuclear signal, we used the *Split by Seed Points* option and set the average diameter of a single cell (in our case to 5µm). This step will allow creating seeds for each single cell within the segmented surface that will be used as a reference point to separate touching cells using a watershed algorithm downstream.

d. Set a threshold for the *Filter Cell Seed Points* function: We established a threshold based on the quality measurement (fluorescence intensity at the center of the object). Other parameters could be taken into account, such as maximum/mean/median/minimum intensity. At this stage, it is helpful to temporarily deselect the rendering of the cell surface (in the *Settings* tab) so that the seed for all individual cells can be evaluated and the threshold adjusted accordingly (the surface rendering is not transparent and hides the cell seed points).

e. Set a threshold for the *Filter Cell* function: here, we filtered out cells based on size only (*Number of voxels*), but other parameters such as distance to neighbors, sphericity, distance to border, volume, or sphericity could also be used.

f. Segmenting the *Spots* (RNAscope signals) within segmented *Cell Surfaces*. Select the channel used for spot segmentation, configure the average spot size (*Estimated Diameter*), and select options for *Background Subtraction* and variable size spots [*Different Vesicle Size (Region Growing)*]. At this stage, several types of spots can be segmented simultaneously (for multiplexed RNAscope experiments with signals in different channels, for example).

g. Set a threshold for the *Filter Vesicles* function: We established a threshold based on the quality measurement (fluorescence intensity at the center of the object), but other parameters could be used alone or in combination. This generates a unique seed for all Spots, and all seeds have the same size at this step.

h. Configure the *Vesicle Region Growing* function. This function takes the seeds generated in the previous steps and expands or reduces their volume according to the size of the RNAscope fluorescence signal.

4. Once the *Cell* objects are generated, segmentation can be modified manually: merge or split objects, remove artefactual objects (in our example, artefactual cells were detected in the notochord because of its autofluorescence), duplicate, subset, or classify them. The classification can be manually curated (one selects cells and creates several categories; these categories are then usable for further statistical analysis) or automatically generated based on quantifiable parameters: number of spots contained in the cell, intensity of the spots, localization, size… The display of the object (color, transparency) can also be modified: cells can be colored uniformly, randomly, or with a statistic-based code (with the parameter of your choice, such as the ones aforementioned). Each object has an ID number and can be identified when in post-processing analysis.

Imaris allows to save screenshots of images, create movies from 3D rendering (see Videos 2 and 3), and hide and show any layer (e.g., 3D grid, fluorescence channels, objects).

**C. Quantitative analysis**

**Figure 9.**
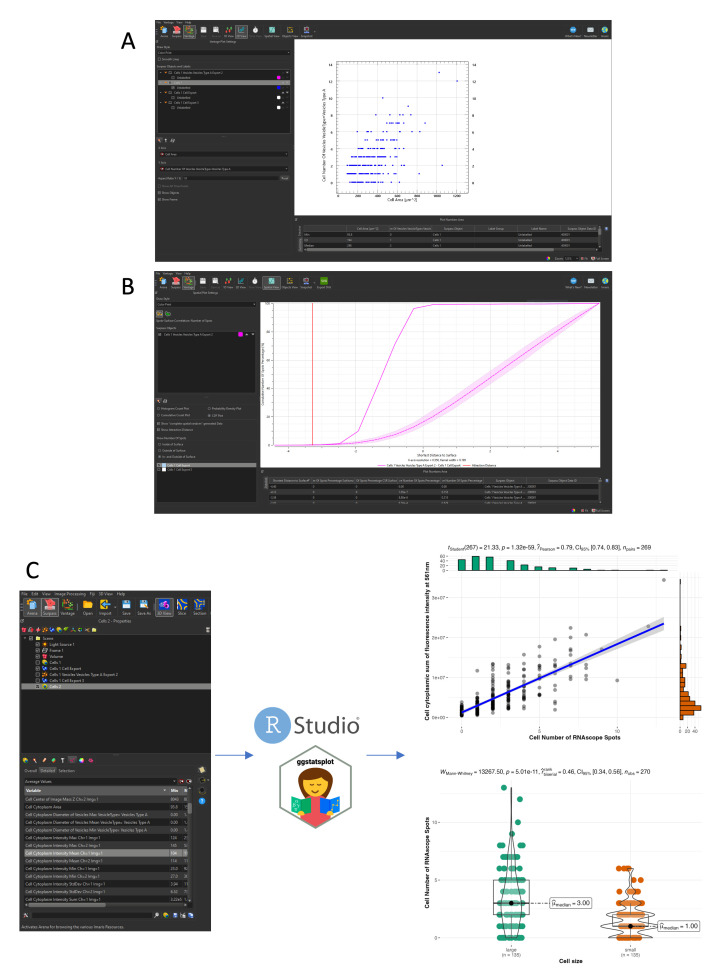
Spatial analysis and plotting of gene expression data. All data plotted are extracted from a single caudal hematopoietic tissue (CHT) z-stack showing the expression of *cmyb* (RNAscope spots) in hematopoietic cells expressing eGFP under the control of the *runx1+23* enhancer. (A) Imaris Vantage 2D plotting, cell number of RNAscope spots relative to the cell area (µm). (B) Imaris vantage cumulative distribution function (CDF) plotting, cumulative number of spots percentage relative to the shortest distance to cell surface (µm). Solid pink line: our data; dashed pink line and light pink interval: confidence interval’s random distribution; red vertical line: calculated attraction distance. (C) Data extraction from Imaris and plotting in R studio using the ggstatsplot package. Top plot shows the correlation between cell size (µm) and number of spots per cell. Bottom plot shows the number of spots per cell depending on cell size [large cell > 11,490 voxels (corresponding to the median cell size), small cells < 11,490 voxels]. The code to generate the plots is given in Data analysis, section C.

1. Imaris proposes a quantitative data visualization module, the *Vantage* mode (Figure 9A), that allows for all objects created in the opened image to generate plots to analyze data.

a. Plots can be *1D view* (boxplots with any variable of interest on the y-axis), scatter plots (*2D View*, with each point characterized by two variables plotted on the x- and y-axis, such as the number of spots as a function of cell size, as shown in Figure 9A), or grid/gallery views (with all segmented objects aligned on a grid and classified depending on the variable that was chosen).

b. The *Spatial View* proposes an analysis of the position of objects [for example, the cumulative number of RNAscope *Spots* as a function of the distance to *Cells* surface in the *Cumulative Distribution Function (CDF)* plot], compares it to a random distribution in space, and interprets it as an attraction or repulsion between objects. In our case (Figure 9B), when looking at spots, the attraction distance is negative (as expected), the data line (solid pink line) is higher than the confidence interval’s random distribution (dashed pink line and light pink interval), and almost 100% of points are located within a distance of 0 µm from the cell surface.

2. Imaris only plots data for one image and, to compare data from multiple embryonic regions or multiple replicates, all data regarding spatial, fluorescence, geometrical, and interaction features can be saved in .csv or .xls files (*Statistical tab*, Figure 9C left) for downstream analysis using alternative data analysis software, such as R, Prism (which requires a license), or Matplotlib. We used RStudio and specific packages [23,24] to read the data (multiple files in a row), perform statistical analysis, and generate plots for publication (Figure 9C, right). Below are code snippets for data reading and plotting. More information on the use of the ggstatsplot package can be found on https://indrajeetpatil.github.io/ggstatsplot/ .

a. R library setup

library(stringr)

library(dplyr)

library(ggplot2)

library(ggstatsplot)

library(readxl)

b. Read multiple .xls files generated by Imaris

# set you working directory

setwd("your path")

# list all .xls files save from Imaris

ls<-list.files("your path/", pattern = ".xls")

# create an empty dataframe were data will be store

df<-data.frame()

# function to read all listed files automatically

for (i in 1:length(ls)) {

# print the name of the file currently being read

 print(ls[i])

# read the sheet of interest in the .xls file you are reading (here for example the sheet corresponding to the number of vesicle per cell) and create a dataframe containing all information from this sheet

 temp<-read_excel(ls[i], sheet = "Cell Number Of Vesicles Ves-2",

 col_names = TRUE, skip = 1)

# add a “file” column to your dataframe containing the name of the file you read. This information is important when reading multiple files, as it allows to retrieve information on the origin of your data after having merged everything together

 temp$file<-as.character(substr(ls[i], start = x, stop = y))

# same as above, allows you to read multiple sheets from the same file depending on the information you want (here two example, the sheet with information on cell size [number of voxel], and the sheet with information on fluorescence signal in the RNAscope channel

 temp2<-read_excel(ls[i], sheet = "Cell Number of Voxels",

 col_names = TRUE, skip = 1)

 temp3<-read_excel(ls[i], sheet = "Cell Intensity Sum Ch=2 Img=1",

 col_names = TRUE, skip = 1)

# merge the columns of all temporary dataframes generated, the column used for merging is “ID”, that corresponds to the unique ID that identifies all Cell objects.

 temp<-merge(temp, temp2, by="ID", all.x = TRUE)

 temp<-merge(temp, temp3, by="ID", all.x = TRUE)

# at each loop of the function, append the new opened data to the dataframe containing previously opened data

 df<-rbind(df, temp)

}

c. Generate plots with ggstatsplot package

# generate boxplot with automated statistical comparisons between several conditions (here non-parametrical testing).

ggbetweenstats(data=df, x=size, y=`Cell Number Of Vesicles`, type="nonparametric", pairwise_comparisons=TRUE, pairwise.display = "none",

 point.args = list(position = ggplot2::position_jitterdodge(dodge.width = 0.5), alpha = 2, size=4 ),

 centrality.point.args = list(color="black", size=3),

 centrality.label.args = list(nudge_x = 0.5, segment.linetype = 4,min.segment.length = 0),

 results.subtitle = TRUE,

 ggplot.component = list(labs(title="", x="Cell size", y="Cell Number of RNAscope Spots")))

# generate scatterplots to identify correlations between two variables, with automated correlation coefficient calculation.

ggscatterstats(

 data = df,

 x = `Cell Number Of Vesicles`,

 y = `Cell Intensity Sum`,

 xlab = "Cell Number of RNAscope Spots",

 ylab = "Cell cytoplasmic sum of fluorescence intensity at 561nm",

 title = ""

)

## Validation of protocol

This protocol was validated for embryos in [8]. For its translation to the 5 dpf larvae, see [15].

In [8], the protocol applied to 48 hpf embryos was used in Figure 3/figure supplement 1A (with the *cmyb* probe) and Figure 4 (with the *pard3ba* probe); Figure 3/figure supplement 2 (with the *pard3aa, pard3ba*, and *pard3ab* probes in panels A, B, and C, respectively) shows large fields of the entire body region at the level of the trunk and encompassing the peripheral nervous system (spinal cord), the notochord, the dorsal artery, the vein, and the gut region; Figure 3/figure supplement 2D shows magnifications of aortic regions to visualize RNAscope dots for the three *pard3* probes at the cellular level; Figure 3/figure supplement 3B (with the pard3ba probe) shows aortic regions; and Figure 3/figure supplement 4 (with the *pard3aa* and *pard3ab* probes in panels A and B, respectively) shows magnifications of aortic regions.

In [15], the protocol was applied to 5 dpf larvae using *gata2b* (ACD Biotechne, catalog number: 551191-C2), *cd34/podocalyxin* (ACD Biotechne, catalog number: 1223761-C1), *cmyb* (ACD Biotechne, catalog numbers: 558291-C1, 558291-C2, 558291-C3), and *timp4.2* (ACD Biotechne catalog number: 1255031-C3) probes.

## General notes and troubleshooting

**General notes**

1. The enzymatic digestion of the biological material is critical, particularly when the mRNAs of interest are expressed in deep tissues in animals (e.g., the periglomerular region in the larva here; see Figure 5). The efficiency of enzymatic batches may also be subject to change. Of note, the Multiplex Fluorescent Reagent kit v2 contains a set of proteases (information not provided by the supplier) that can be tested, in addition to the proteinase K used in this protocol. Testing these proteases may be useful for other stages, organs, and animals.

2. Bio-techne is now providing a reagent kit with new TSA (tyramide signal amplification) Vivid^TM^ dyes that provide brighter signals [TSA Vivid dyes 520 (green), 570 (orange), and 650 (red)]. These dyes are suitable for multiplexing (three probes concomitantly), and their optical properties are compatible with the use of DAPI for staining of nuclei. The combination of detection of three mRNAs and one protein target using antibodies is also possible.

**Troubleshooting**

1. A major problem for zebrafish embryos and larvae is that the RNAscope wash buffer (WB) is harsh; consequently, the animals become translucent, smooth, and easily damaged. The advantage of the harsh buffer is that it limits background; this is an essential parameter. If necessary, washing steps can be optimized by diluting the WB or using an alternative buffer [e.g., SSC buffer at different concentrations (UltraPure 20×, Invitrogen, catalog number: 15557-044)]. Because animals become smooth, centrifugation should be avoided, and animals should be sedimented between washes only by gravitation. The removal of any medium (WB and media with reagents) should be, at all steps, monitored under the binoculars.

Note also that regarding the morphology of animals (which is more critical for embryos), an alternative that is faster and probably less damaging is to use the HiPlex assay (https://acdbio.com/rnascope-hiplex-assays), with which three fluorophores can be added together directly after HiPlex AMPs (1–3). The limitation of using HiPlex is its cost, which is approximately twice the price of the Multiplex assay and which uses different probes.

2. The reaction gives large patches and not individual spots (e.g., Figure 5), which does not occur in the majority of cases; this precludes quantification of dots. Probes should not be diluted (recommendation from the supplier). One step that may be worked on is the incubation time and temperature of the dye coupling in TSA buffer (this parameter was not tested). If the information is critical, the user may contact the supplier to reduce the number of ZZ pairs. Another alternative may be to expand the tissue to decrease the density of the signal, for example by using expansion microscopy; for one such example in the zebrafish larva, see [25,26]. Still, the translation of RNAscope to expansion conditions needs to be investigated; see [27].
